# Supplementary material for: The causal relationship between the human gut microbiota and pyogenic arthritis: a Mendelian randomization study
Source: Front Cell Infect Microbiol. 2024 Nov 26;14:1452480. doi: 10.3389/fcimb.2024.1452480 (PMC11629706; doi:10.3389/fcimb.2024.1452480)
Supplement: Supplementary file 1 [file DataSheet1.zip › mendelian test/ebi-a-GCST90027647.csv_forest.pdf]

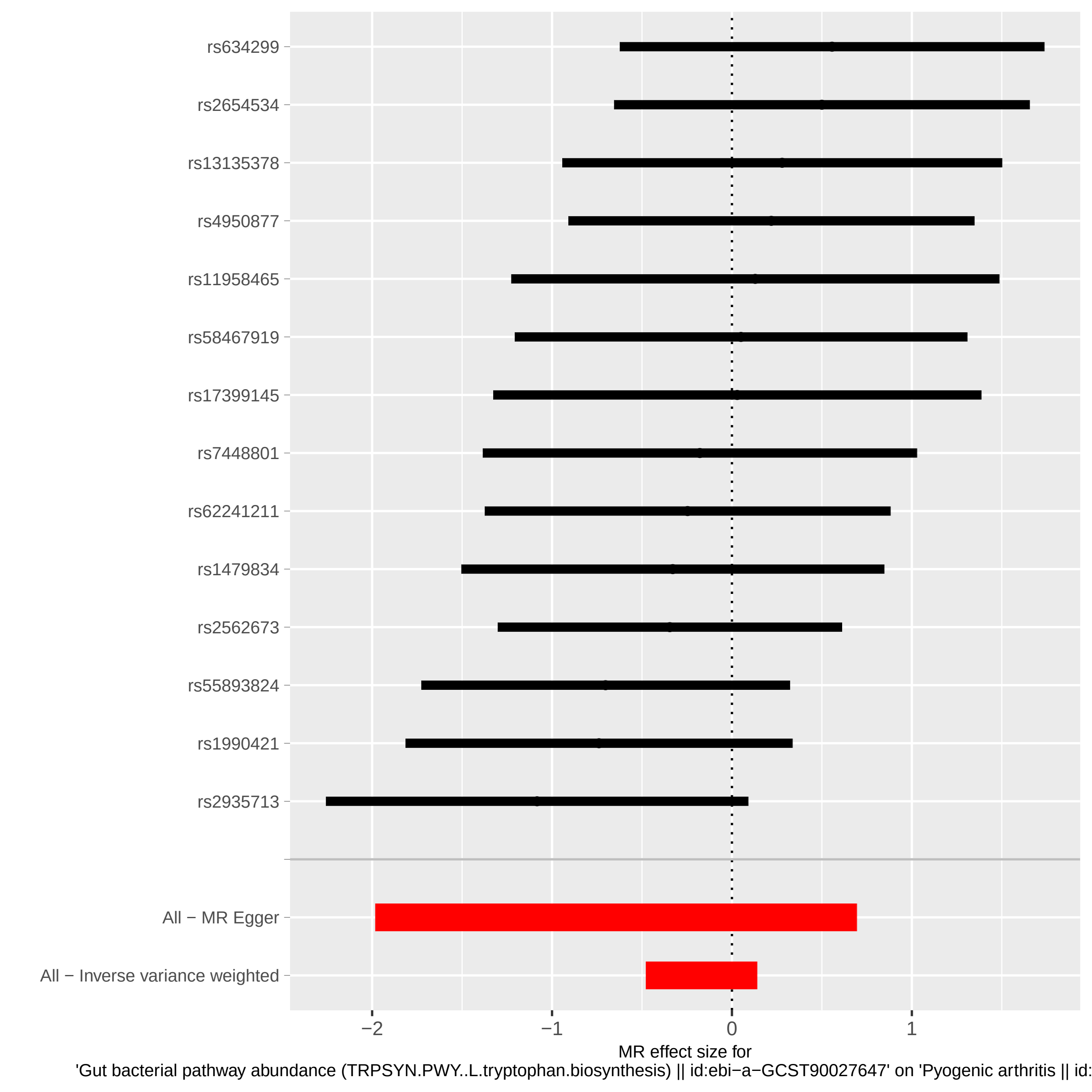
rs634299
rs2654534
rs13135378
rs4950877
rs11958465
rs58467919
rs17399145
rs7448801
rs62241211
rs1479834
rs2562673
rs55893824
rs1990421
rs2935713
All − MR Egger
All − Inverse variance weighted
−2
−1
0
1
MR effect size for
'Gut bacterial pathway abundance (TRPSYN.PWY..L.tryptophan.biosynthesis) || id:ebi−a−GCST90027647' on 'Pyogenic arthritis || id:
